# Unusual Presentation of an Ileal Duplication Cyst: A Case Report

**DOI:** 10.7759/cureus.38959

**Published:** 2023-05-13

**Authors:** Nadir Miry, Nassira Karich, Mohammed Bakhti, Tarik Deflaoui, Amal Bennani

**Affiliations:** 1 Department of Pathology, Mohammed VI University Hospital, Oujda, MAR; 2 Department of Visceral Surgery and Digestive Oncology, Mohammed VI University Hospital, Oujda, MAR

**Keywords:** digestive tract malformations, midgut duplication cyst, ileal duplication cyst, respiratory epithelium, duplication cyst

## Abstract

Duplication cyst (DC) of the digestive tract is a rare embryological anomaly, presenting as a cystic formation that could be attached to any part of the digestive tract, it is a thin-walled structure made of two layers, an inner layer that is frequently lined by an alimentary epithelium, surrounded by a smooth muscle layer often shared with the adjacent digestive segment. DCs are most commonly located in the distal ileum; sometimes, they are associated with other visceral or skeletal anomalies. They are frequently discovered during childhood, following a bowel obstruction or abdominal pain.

Here we report a rare case of an ileal DC lined by a pseudostratified and ciliated epithelium, discovered in an adult patient following intestinal obstruction syndrome.

## Introduction

Digestive DC is a rare finding in adult population, especially those lined by a respiratory epithelium, it is characterized by a variable clinical presentation. Sometimes associated with other digestive or extra-digestive defects. Most DCs are discovered during infancy, they rarely stay asymptomatic during childhood and do not manifest until adulthood [1]. DCs could be located at any segment of the digestive tract, most commonly in the ileum; it is frequently lined by an alimentary epithelium, rarely an ectopic lining, such as a respiratory epithelium [2]. Several hypotheses have been suggested to explain the embryological pathogenesis of such abnormality, but seemingly none of these theories is able to provide a clear explanation from an embryological perspective, especially DCs lined by non-alimentary epithelium.

## Case presentation

**Figure 1:**
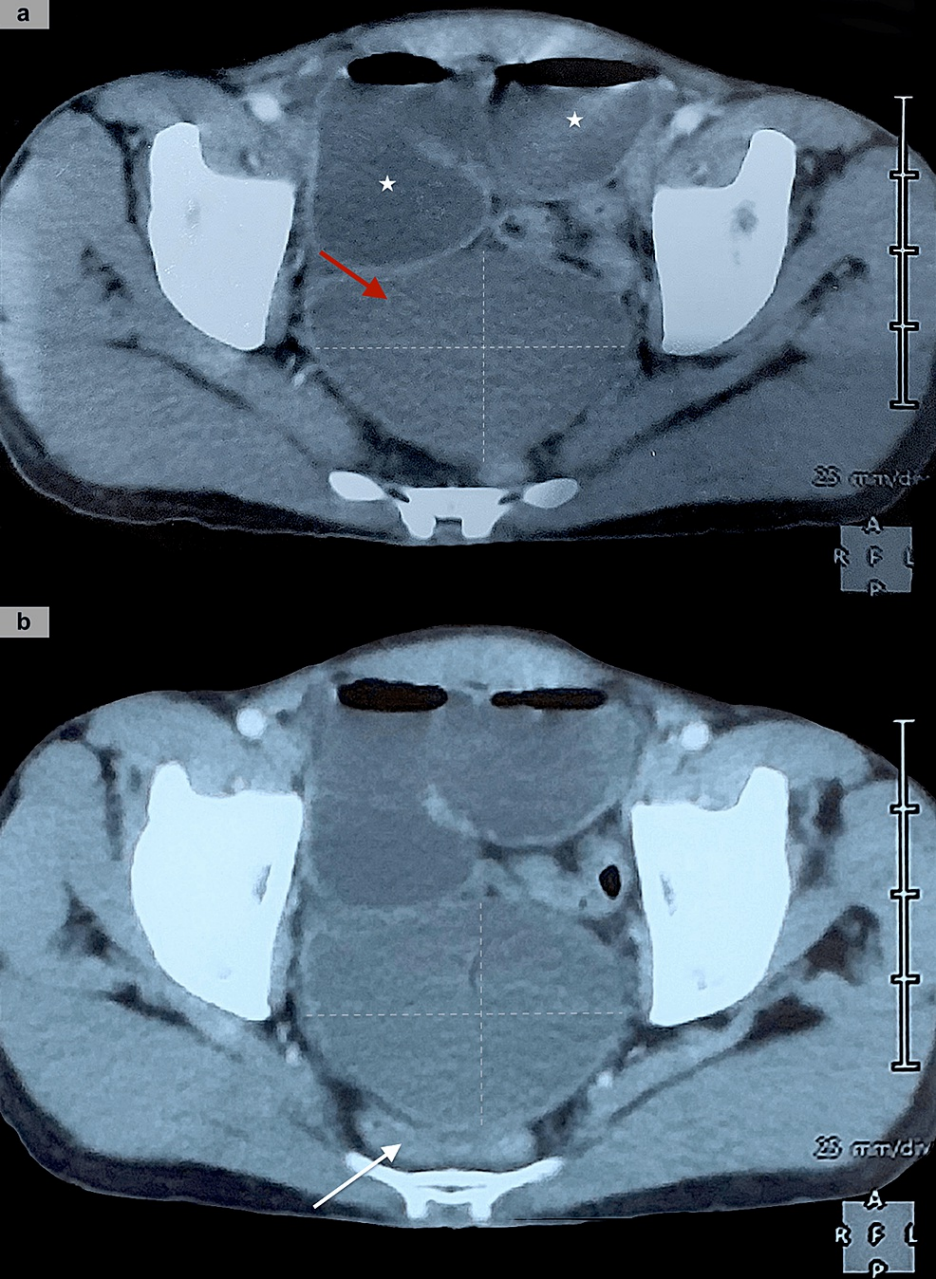
Axial CT scan revealed a well-circumscribed cystic mass (red arrow), located in the pelvis, along with numerous gas-fluid levels in distended bowel loops (white star) (a). The cyst caused a mass effect on the rectum (b).

Here we report a case of a 47-year-old male, with no medical history, who was admitted to the hospital, due to a presentation marked by abdominal pain and swelling, and reported that he had not passed gas or had a bowel movement for four days. Otherwise, he didn't mention any similar episodes in the past, or any other digestive or general symptoms. Abdominal CT scan revealed a large cystic mass measuring 93×71×51 mm, attached to the ileal segment with evident mass effect on the adjacent segment, responsible for an ileal obstruction (Figures 1a, 1b).

No contrast enhancement was observed and no genitourinary or vertebral anomalies were noted. During surgical procedure, the patient underwent a complete resection of the mass along with the attached ileal segment, followed by an ileal anastomosis; there were no signs of associated intestinal atresia or perforation.

Gross examination of the resected segment showed a 10 cm, thin-walled cystic mass filled with mucinous fluid closely attached to a 15 cm long intestinal segment; macroscopic examination of both cystic wall and the normal ileal segment didn’t reveal any signs of perforation, atresia or communication between the cyst and the ileal lumen.

**Figure 2:**
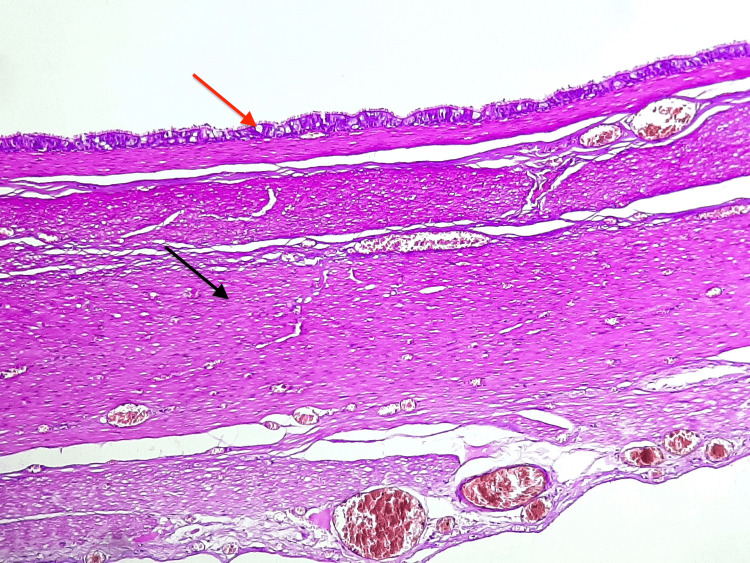
Photomicrograph showed a cystic wall lined by a pseudostratified epithelium (red arrow), surrounded by a second layer of smooth muscle fibers (black arrow) (H&E, 200x).

**Figure 3:**
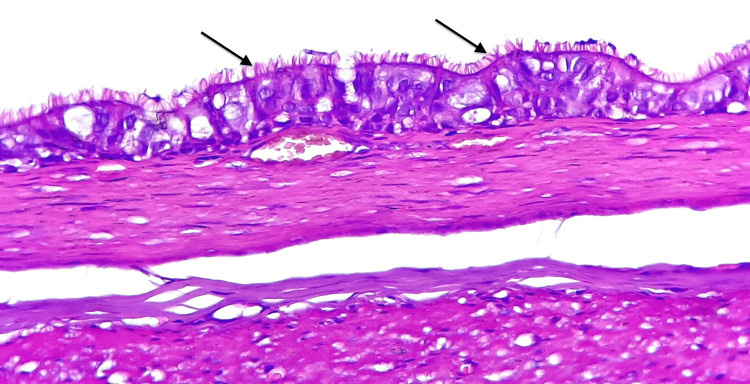
At higher magnification, the epithelial lining is made of a pseudostratified and ciliated epithelium (arrows), with no evident bronchial glands or cartilage (H&E, 400x).

Histological examination of the cyst wall shows a pseudostratified, ciliated columnar epithelium surrounded by a smooth-muscle layer fused and hardly distinguishable from the ileal muscularis propria. No seromucinous glands or cartilage were seen (Figures 2, 3). The particularity of our case resides in both the respiratory nature of the epithelial lining and the late manifestation until adulthood.

## Discussion

Intestinal DCs are rare cystic congenital malformations that communicate rarely with the intestinal lumen [1]. They develop between the fourth and eighth week of gestation, and they rarely cause any trouble antenatally [2]. DC could be found at any segment along the digestive tract from the oral cavity to the anus [3], it occurs most commonly in the distal ileum, followed by the colon, esophagus, stomach then the duodenum [4]. It is responsible for a wide variety of digestive symptoms, depending on its size, location, and the nature of its epithelial lining; clinical manifestations range from mild abdominal pain, nausea, vomiting, and abdominal mass to serious symptoms such as gastrointestinal ulcerations, hemorrhage, or anemia; It could also be revealed by complications including bowel obstruction, perforation, or peritonitis [5]. Most cases are revealed during the first two years [2].

Duplication cysts are subdivided into tubular and cystic types, the latter is more frequently encountered in the small intestine. They can be communicating or non-communicating with the adjacent intestinal segment [6]. Skeletal malformations are frequently associated with foregut DCs, whereas genitourinary and other gastrointestinal anomalies are associated with midgut and hindgut DCs [5]. Duplication cyst in our case was non-communicating to the adjacent ileal segment, with no evident skeletal or genitourinary deformities.

Histologically, the cyst wall is lined, most commonly, by an alimentary epithelium similar to the adjacent digestive segment or rarely a heterotopic lining that differs from the adjacent segment, and exceptionally an extra digestive lining, such as a respiratory epithelium, as in our case; it is also possible to come across an ectopic pancreatic tissue within the cyst wall [7]. The rest of the cyst is composed of a smooth muscle layer, most often shared with the adjacent digestive segment [6]. The presence of a gastric-type mucosa is frequently associated with ulcerations and perforations, due to acidic secretions, which explains early manifestations in such cases [8]. Thus, the late onset in our case could be explained by the absence of any gastric or pancreatic tissue that could be responsible for mucosal irritation, ulceration, or perforation.

Many theories have been proposed to explain digestive DCs including aberrant recanalization and partial twinning, which explain the presence of DCs in both esophageal and anorectal locations and their association to genitourinary tract anomalies. Persistent diverticula theory explains small intestine DCs. The split notochord theory explains associated spinal anomalies with enteric DCs [9]. Lastly, abnormal intrauterine vascularization explains DCs associated with small bowel atresia [7].

Most DCs with a respiratory lining are located in the foregut (oral or esophageal segment) [9], the presence of such ectopic epithelium at a proximal location is explained embryologically by an abnormal separation of the esophagus and trachea during embryological development [10]. Respiratory lined DCs in distal locations are extremely rare, and sometimes misdiagnosed as midgut or intestinal bronchogenic cysts which are, by definition, defects of the respiratory tract.

Only three cases of respiratory lined DCs of the ileum were reported, two belonged to pediatric population and only one case was an adult patient which contained an admixture of squamous, intestinal, and respiratory lining [11,12]. The case reported here is the only known case of an ileal DC with pure respiratory lining in an adult patient. Intestinal DCs are treated surgically by resecting the cystic formation along with the adjacent intestinal segment followed by an anastomosis [6].

## Conclusions

Digestive DCs are rare developmental abnormalities, affecting the pediatric population more frequently, they are most commonly located in the distal intestine and usually lined by an alimentary epithelium; the presence of a respiratory lining is exceptional, especially in distal locations. The present case highlights both the delayed onset of digestive symptoms and also the presence of non-alimentary epithelium in intestinal DCs.
